# Atypical Presentation of Paget-Schroetter Syndrome: Case Report and Management

**DOI:** 10.7759/cureus.25424

**Published:** 2022-05-28

**Authors:** Jaydip Desai, Arsh N Patel, Sammy Dahan, Fulton Defour

**Affiliations:** 1 Department of Research, Alabama College of Osteopathic Medicine, Dothan, USA; 2 Department of Internal Medicine, Thomas Hospital, Fairhope, USA

**Keywords:** thoracic outlet syndrome, internal medicine, effort thrombosis, orthopedic surgery, peripheral vascular surgery, deep vein thrombosis (dvt)

## Abstract

Upper extremity deep vein thrombosis (UEDVT) is a rare condition that makes early clinical evaluation and treatment important prior to the formation of deep vein thrombosis (DVT). Typical risk factors include male sex, young age, repetitive arm over abduction and hyperextension, indwelling catheters, cervical first rib, and thoracic outlet syndrome. One common cause of UEDVT is Paget-Schroetter syndrome (PSS). If untreated, pulmonary complications such as venous thromboembolic disease and pulmonary embolism (PE) may develop. We present a case of a 34-year-old Caucasian female who presented to the emergency department with sudden, acute right arm pain after blow-drying her hair, consistent with UEDVT. CT angiography (CTA) demonstrated moderate thromboembolic disease within segmental and subsegmental branches of the left upper, left lower, and right lower lobes. Ultrasonography (US) of the upper extremity showed non-compressibility of the right axillary and basilic vein, a finding consistent with acute DVT. Peripheral angiogram revealed imaging consistent with undiagnosed thoracic outlet syndrome secondary to effort thrombosis. The patient deferred surgical intervention and agreed to begin long-term anticoagulation therapy. PSS requires immediate recognition and treatment to prevent possible long-term neurologic and vascular compromise. Despite the patient lacking the typical population demographics, PSS should be considered given the patient's symptoms and presentation. Recognition of UEDVT despite classic signs and symptoms consistent with known risk factors is imperative upon clinical suspicion. Delay in clinical management may lead to fatal complications. We aim to highlight a case of PSS along with alternative pathways for treatment delivery.

## Introduction

Upper extremity deep vein thrombosis (UEDVT) is defined as thrombus formation that occurs in the axillary, subclavian, radial, ulnar, or brachial veins in the upper extremity. It is less common than lower extremity deep vein thrombosis (LEDVT), with an annual incidence of two out of every 100,000 cases, accounting for 1-4% of deep vein thrombosis (DVT) formation [1]. Acute UEDVT formation presents clinically with upper extremity arm pain and skin changes. However, swelling and arm discomfort have been the most reported common symptoms in patients with UEDVT. Because UEDVT symptom specificity on physical examination has a poor representation, immediate clinical intervention is warranted if UEDVT is suspected. Diagnosis may be made with compression ultrasonography as the initial, preferred diagnostic test due to its high sensitivity and specificity [1]. However, CT or magnetic resonance (MR) venography may be able to define upper extremity anatomy clearer than ultrasound and is considered the gold standard for diagnosis. Treatment is focused on anticoagulation for three to six months, and if contraindicated, percutaneous superior vena cava filter placement is considered.

The common causes of UEDVT include Paget-Schroetter syndrome (PSS), also known as effort thrombosis. This condition is defined as incidental trauma due to muscular strain, making up anywhere between 20% and 40% of UEDVT [2]. Other causes, specifically in the elderly cohort, include central venous catheter placement, ports, pacemakers, and occult malignancy. The incidence ranges from one to two per 100,000 individuals per year [2]. The proposed mechanism of pathogenesis behind PSS occurs when the repetitive motion of retroversion, hyperabduction, and extension of the arm leads to increased damage to the walls of the various veins involved, leading to fibrosis [2]. As a result, the coagulation cascade becomes activated with venous stasis. The repetition of specific motions creates a cycle of thrombosis and recanalization. Mixed studies have demonstrated that PSS and idiopathic UDEVT has been associated with factor V Leiden and prothrombin gene mutation, while others have shown an unclear linkage between UEDVT formation and thrombophilic disorders.

Identified risk factors for PSS include young age, male sex thrombophilia, repetitive overarm hyperabduction, and thoracic outlet anatomical abnormalities [3]. Some factors, such as thrombophilia, play an evident and major role in the pathogenesis of thrombus formation. Other more insidious factors in regards to the anatomical network adjacent to the thoracic outlet include cervical first rib, hypertrophy of the anterior scalene, abnormal tendinous insertions, or even acquired variations such as overgrowth due to bony trauma. These structural components contribute to pathogenesis by compromising linearity and contributing to turbulent blood flow, resulting in thrombus formation. Subsequent decompression of the venous thoracic outlet, with long-term anticoagulation, is often viewed as a necessary intervention by means of excision of the first rib, partial anterior scalenectomy, or division of any other anatomic anomalies that may compress local vasculature to prevent a recurrence.

We present a case of risk factors in a middle-aged woman with minimal to no repetitive overarm hyperabduction that led to the spontaneous development of UEDVT with undiagnosed thoracic outlet syndrome, leading to pulmonary sequelae of thromboembolic disease. Our goal is to increase awareness of the variety of risk factors that lead to PSS and highlight the clinical consequences if not properly intervened, as well as possible treatment algorithms.

## Case presentation

A 34-year-old Caucasian female presented to the emergency department with right arm pain. It started suddenly while she was straightening her hair in the bicep region prior to going to work. Her pain was described as “pinching” and she noticed discoloration that mimicked a bruise. The patient denied any recent falls or trauma to the area. She had no personal or family history of clotting disorders and had not used any birth control in the past three months. Additionally, she claimed low activity levels and denied any repetitive overhead activities. However, she does report smoking one pack per day for about 13 years.

On physical exam, she was noted to have swelling and tenderness to the right upper arm extending to her shoulder. Ecchymosis was appreciated on the right medial portion of her biceps. She had palpable radial and ulnar pulses with warm and dry skin, and a normal range of motion of the shoulder joints bilaterally.

**Figure 1:**
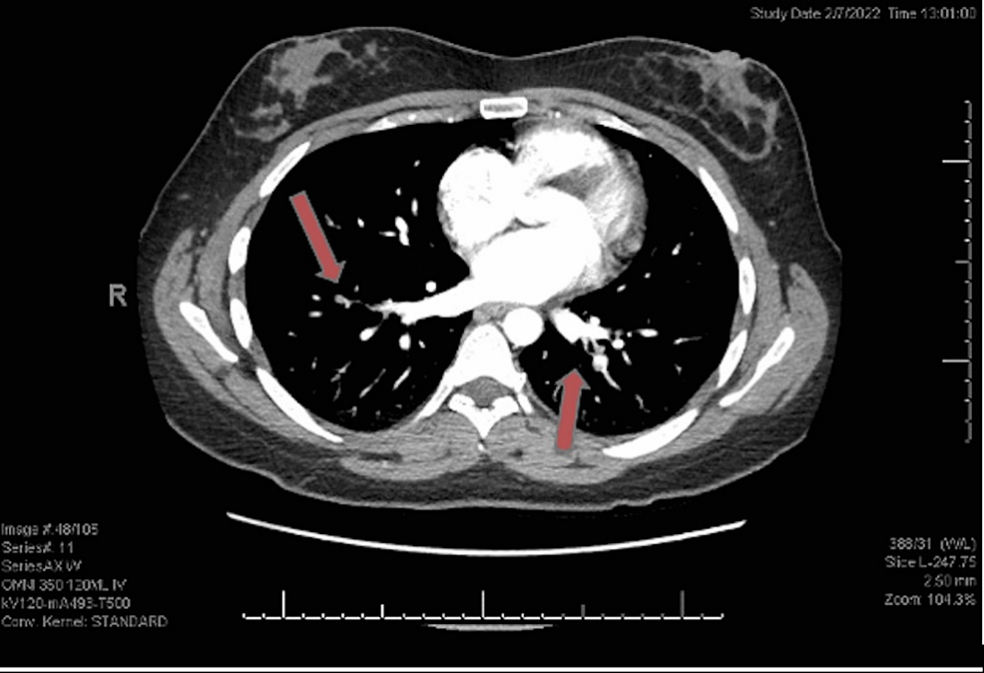
CT angiography of the chest with contrast illustrating multiple lobular infarcts consistent with thromboembolic disease. The arrows represent areas of pulmonary infarction caused by upper extremity deep vein thrombosis.

**Figure 2:**
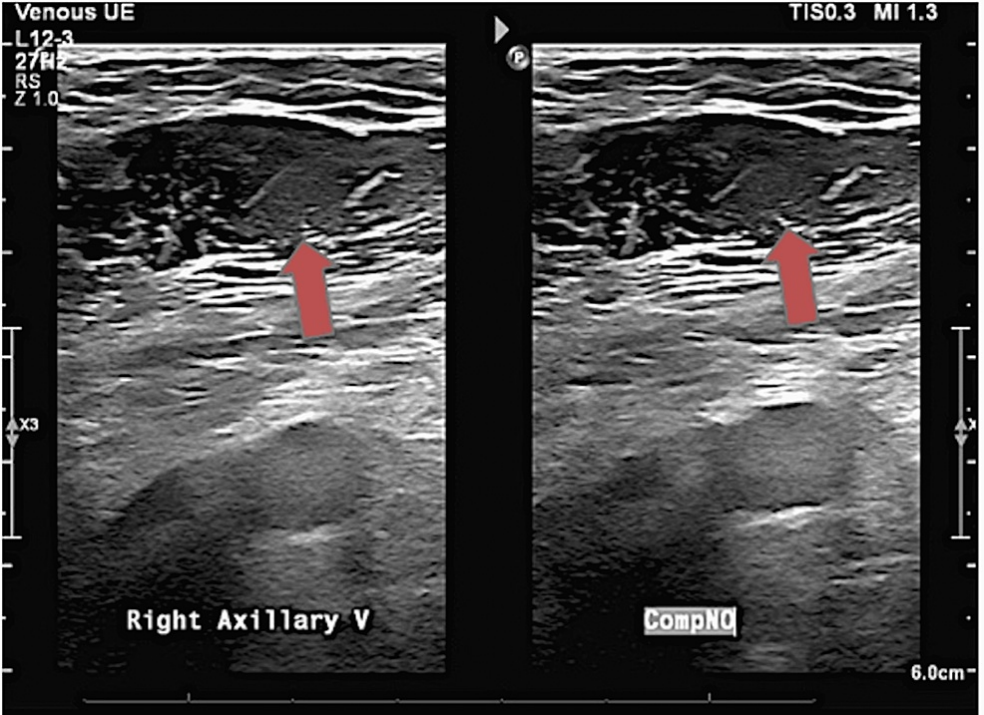
Compression ultrasonography (US) of the upper extremity illustrating thrombus formation in the right basilic and axillary vein. The arrows in the US represent about 60% of total thrombus formation in the vessel lumen of the axillary vein.

CT angiography (CTA) ordered in the emergency department showed moderate thromboembolic disease within segmental and subsegmental branches of the left upper, left lower, and right lower lobes (Figure 1). No thrombus was seen in the outflow tract or main pulmonary artery, but evidence of mild right heart strain was observed. Ultrasonography of the upper extremity showed non-compressibility of the right axillary and basilic vein, a finding consistent with acute UEDVT (Figures 1, 2). When discussing the results, the patient stated she did not have any chest pain or shortness of breath prior to arrival. The patient was started on a heparin drip and admitted to the floor for medical management.

**Figure 3:**
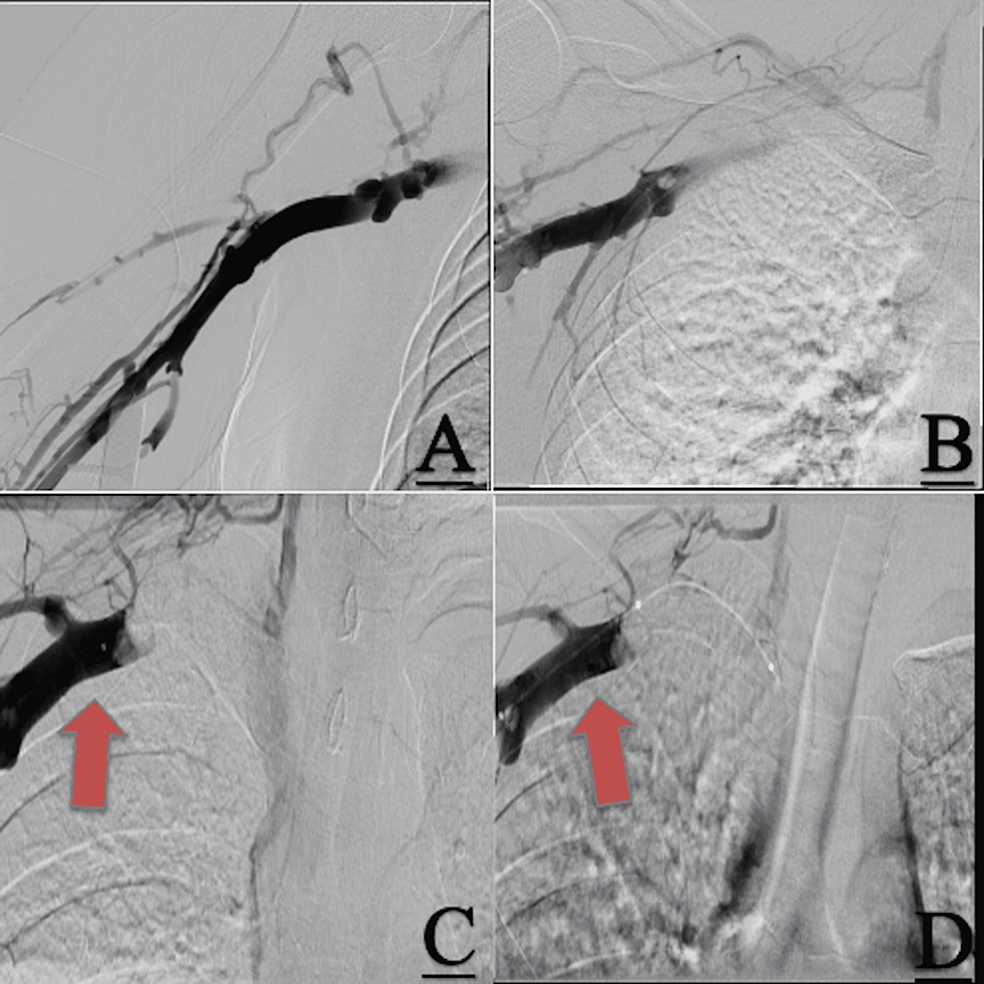
Angiogram demonstrating thoracic outlet syndrome. A-D represent the transition of contrast injected and traveling from the distal extremity to the proximal. C and D demonstrate the contrast passing in the subclavian artery passing through and under the clavicle, indicative of thoracic outlet syndrome.

Coagulation studies for protein C/S, anti-thrombin III, factor V Leiden, and PT20210A were all deemed to be negative. She was then scheduled for a peripheral angiogram per multi-disciplinary recommendations after thrombus resolution was noted. The patient appeared to have thoracic outlet syndrome, with the right axillary vein compressed by the clavicle (Figure 3). Vascular surgery was consulted regarding surgical intervention. The patient ultimately agreed to continue long-term anticoagulation and agreed to begin smoking cessation.

## Discussion

UEDVT makes up a minor portion of DVT formation in the body, accounting for 1-4% of thrombosis. Furthermore, PSS accounts for a smaller population of approximately 20-40% of those with UEDVT. The rare incidence of this condition makes early diagnosis and treatment pivotal for symptomatic relief along with positive long-term patient outcomes. Mintz and Levy share that 5-8% of UEDVT present with clinical pulmonary embolism (PE), while subclinical PE accounts for close to 36% of suspected cases [4]. Often cases presented in the literature involve patients impacted by PSS due to repetitive injuries, such as overhead athletics, poor sleeping posture, working in an assembly line, and painting. Bushnell et al. termed such occurrences as “effort thrombosis,” which signifies a forceful event that produces direct or indirect injury to the vein [5]. Early diagnostic maneuvers such as Adson and Wright tests done in conjunction with clinical symptoms should raise suspicion of thoracic outlet syndrome [5]. Adson test can help diagnose subclavian vessel entrapment between the anterior and posterior scalene, while Wright’s maneuver is used to screen for vessel entrapment between the subcoracoid region and pectoralis minor. Currently, the gold standard for diagnostic screening is direct contrast venography for thrombosis in the upper extremity followed by angiography for PE.

**Figure 4:**
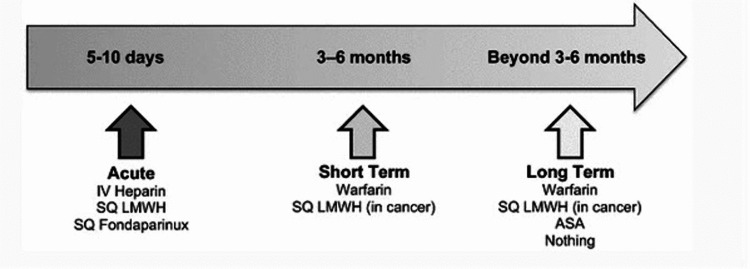
Recommended long-term anticoagulation therapy for venous thromboembolism presented by Streiff et al. Source: Streiff et al. (2016) [6]. License: The original article is distributed under the terms of the Creative Commons Attribution 4.0 International License (http://creativecommons.org/licenses/by/4.0/), which permits unrestricted use, distribution, and reproduction in any medium, provided you give appropriate credit to the original author(s) and the source, provide a link to the Creative Commons license, and indicate if changes were made*. * Permission was obtained and no changes were made from the original image. SQ: subcutaneous; LMWH: low molecular weight heparin; ASA: aspirin.

For the right patient, therapy aimed at early catheter intravenous thrombolysis followed by heparin-warfarin bridge therapy to achieve long-term anticoagulation therapy has shown to have beneficial patient outcomes (Figure 4) [6]. Long-term conservative treatment should be focused on outpatient rehabilitation with physical therapy to improve pain, edema, and ergonomics to prevent reoccurrence. A recent study at a metropolitan hospital analyzed the presentation and incidence of different variations of thoracic outlet syndrome whether they be neurological thoracic outlet syndrome (NTOS), arterial thoracic outlet syndrome (ATOS), or venous thoracic outlet syndrome (VTOS), and found that 84% of referred patients presented with symptoms suggestive of NTOS. This suggests that a history of neurologic findings such as paresthesia or weakness and demonstration of abnormal thoracic outlet anatomy should warrant vigilance for complications indicative of thrombosis [7]. However, optimal therapy and the best outcomes have shown support for the combination using thrombolysis and thoracic outlet decompression in up to 90% of patients [8]. Despite the high success rate, invasive therapies should only be considered for severe presentations of thoracic outlet syndrome to reduce recurrence rates as they are already elevated without anticoagulation management [8,9]. In acute events, efforts should be made to conservatively treat the patient with attempts to modify lifestyle or minimize hypercoagulable states to reduce thrombosis recurrence.

## Conclusions

PSS requires immediate recognition and treatment to prevent possible long-term neurologic and vascular compromise. Despite the patient lacking typical population demographics, PSS should be considered given the patient's symptoms and presentation. Further diagnostic evaluation should be done using inexpensive exams such as manual maneuvers and compression ultrasonography. The majority of cases with PSS may lead to subclinical PE; thus, conservative management as outlined in this case report should be considered for the right patient.
